# Morphological Investigation of Uncharacterised Cardiovascular Structures in Shallow-Diving, Semi-Aquatic Freshwater Turtles (Chelidae: *Emydura macquarii*)

**DOI:** 10.3390/vetsci13050493

**Published:** 2026-05-19

**Authors:** Rhiannon Jade Gurkin, Cleide Spronhle-Barrera, Lawrence Noble, Nate Maisel, Jo Gordon, Christopher Lam, Andrea Schaffer-White, Francesco Origgi, Viviana Gonzalez-Astudillo

**Affiliations:** 1 The University of Queensland, School of Agriculture and Food Sustainability, Gatton, QLD 4343, Australia; r.gurkin@uq.edu.au; 2 IDEXX Laboratories Pty Ltd., 10 Brandl St., Eight Mile Plains, Brisbane, QLD 4113, Australia; cleide-sprohnle-barrera@idexx.com; 3 The University of Queensland, School of Veterinary Sciences, Gatton, QLD 4343, Australia; l.noble@uq.edu.au (L.N.); natemaisel96@gmail.com (N.M.); gordonjo158@gmail.com (J.G.); 4 Veterinary Cardiologists Australia, Underwood, QLD 4119, Australia; clam@vcacardiology.com.au; 5 Independent Veterinary Pathology, Underwood, QLD 4119, Australia; andrea.schafferwhite@ivpath.com.au; 6 University of Messina, Department of Veterinary Science, Polo Universitario dell’Annunziata, 98168 Messina, Italy; francesco.origgi@supsi.ch; 7 Institute of Microbiology, Department of Environment, Construction and Design (DACD), 6850 Mendrisio, Switzerland; 8 University of Applied Sciences and Arts of Southern Switzerland (SUPSI), Institute of Microbiology, 6501 Bellinzona, Switzerland

**Keywords:** heart anatomy, cardiac function, diving physiology, atrial smooth muscle, haemodynamics

## Abstract

Reptiles with diving capabilities have evolved physiological adaptations as well as conformational changes to temporarily sustain life underwater. Despite the importance of both respiratory and cardiovascular systems during diving, most studies have focused on respiratory adaptations. Thus, characterisation of previously undescribed cardiovascular anatomical variations in diving vertebrates is of broad interest. Thirteen clinically healthy, free-ranging adult female Murray River turtles (Chelidae: *Emydura macquarii*) were collected for research purposes, euthanised and autopsied. Prominent, valve-like structures, comprised exclusively of smooth muscle myocytes, were identified in medium- and large-calibre muscular arteries of all individuals. Additionally, multiple intramural vascular channels, mimicking post-thrombotic recanalization, were observed within medium-calibre muscular arteries. Further, we confirmed the presence of alpha-smooth-muscle actin-positive cells lining the cardiac atria in *E. macquarii*. Quantitative morphometric analyses demonstrated that the valve-like structures frequently occupied a substantial proportion of the vascular lumen, in some cases exceeding 90% luminal narrowing. Their consistent presence across multiple individuals and organ systems supports the interpretation that these are physiological vascular structures rather than artefacts. This study examines the potential physiological and evolutionary roles of these vascular structures, providing a basis for further research into cardiovascular adaptations in vertebrates subjected to postural changes and diving-related haemodynamic challenges.

## Simple Summary

Reptiles, particularly species capable of diving, have developed a range of physiological and anatomical adaptations that enable them to survive for extended periods underwater. This study aimed to characterise previously undescribed features of the cardiovascular system in the Murray River turtle. Thirteen healthy adult females were examined, revealing distinct valve-like structures within muscular arteries, as well as small intramural channel-like formations within arterial walls, suggestive of structural adaptations that may support blood flow under changing physiological conditions. Additionally, smooth muscle cells were confirmed lining cardiac atria, indicating a potential role in regulating cardiac blood movement. Measurements of these valve-like structures showed that they could occupy a large proportion of the blood vessel lumen, in some cases narrowing the vessel by more than 90%. Their consistent presence across multiple turtles and organs, together with the degree of vascular narrowing observed, supports the interpretation that these are true anatomical structures rather than tissue processing artefacts. Collectively, these findings suggest that turtles possess specialised cardiovascular features that may assist in managing fluctuations in blood pressure and circulation during diving. This study broadens current understanding of how vertebrates tolerate low-oxygen environments and provides a foundation for future research into cardiovascular adaptations.

## 1. Introduction

Diving research conducted into phylogenetically diverse vertebrates such as semi-aquatic reptiles and mammals indicates the existence of distinct adaptations, making diving physiology unique among species [1,2,3,4]. Despite evolutionary divergence, all diving species face similar fundamental physiological constraints related to oxygen limitation, gas exchange and circulatory regulation during submergence, leading to adaptive diving responses such as arterial constriction and bradycardia [5,6]. Examining anatomical adaptations of vertebrates holds significance as it implies that diverse taxa, such as reptiles, have evolved other extra-physiological changes to thrive in aquatic environments and this study attempts to unravel more insights into their life history. In particular, the Murray River turtle (*Emydura macquarii*), a freshwater diving reptile, may exhibit unique anatomical variations, making it a valuable model for studying haemodynamic control of cardiovascular function during diving.

*E. macquarii* is a short-necked turtle broadly distributed throughout the Murray–Darling Basin in New South Wales, Queensland, South Australia, and Victoria, Australia [7]. These turtles predominantly inhabit deep, permanent still waterbodies but are also commonly found in permanent streams and rivers, exhibiting limited terrestrial migratory behaviour [7]. Despite limited overland dispersal, populations show considerable genetic and morphological variation across their range [8,9]. Although freshwater Chelid turtles can migrate long distances within aquatic systems, genetic relationships generally reflect hydrological connectivity rather than geographic distance, with higher diversity often confined to isolated catchments such those of the Murray–Darling Basin; these patterns suggest that morphological and genetic divergence among populations may reflect ongoing allopatric speciation processes [9,10].

These turtles are primarily aquatic, yet comparatively little is known about the anatomical features of their cardiovascular system that may support diving physiology. Although aspects of cardiac and vascular anatomy have been described in other Chelid turtles, much of the extra-cardiac vasculature, including the arrangement of arteries, veins, and valve-like structures, remains poorly characterised, despite previous descriptions of the renal–portal system and associated venous and valvular structures in reptiles [11].

Increasing attention has been given to the application of physiological data for diagnosing reptile cardiac disease in recent cardiovascular research [12,13]. Novel vascular structures, such as the vertebral venous plexus facilitating cerebral perfusion in climbing snakes, have also been described, highlighting persistent gaps in reptile cardiovascular anatomy [14]. Despite this, current understanding of extra-cardiac vascular anatomy in reptiles remains broadly similar across taxa, with only minor species-specific variations, particularly in vessel orientation [15,16].

The aim of this study was to characterise the vascular anatomy of *E. macquarii*, with particular focus on arterial structures that may influence haemodynamic control during diving. By integrating comparative anatomical observations with existing knowledge from other reptiles and vertebrates, we sought to identify anatomical features that may contribute to cardiovascular regulation in freshwater turtles.

## 2. Materials and Methods

A total of 13 wild *E. macquarii* adult female turtles were obtained opportunistically from the secondary use of carcasses. These were obtained for another study conducted as per the Queensland Department of Environment and Science (DES) Wildlife and Threatened Species animal population dynamics survey (AEC permit SA 2018/11/663). Turtles were captured from three different freshwater reservoirs across Queensland as outlined in previous studies [17,18,19,20,21], during the Australian springtime in 2021, with high- and low-temperature variations ranging between 24 and 28 °C and 14 and 19 °C, respectively. Prior to euthanasia, a complete physical exam was conducted including blood collection for clinical pathology. Gross post-mortem assessment was conducted followed by representative histology of major organ systems, including brain, skin, appendicular skeletal muscle, thymus, major arteries, pharynx, trachea, lungs, heart, oesophagus, stomach, intestine, pancreas, spleen, kidneys, and reproductive tract (ovaries and uterine tube).

Samples were fixed in 10% neutral buffered formalin for up to 7 days and processed routinely to produce 4 µm thick, haematoxylin and eosin-stained (H&E) sections. Histochemistry (Masson’s trichrome, Elastin Verhoeff–van Gieson) was performed using established laboratory protocols [22]. Positive and negative histochemistry controls for Masson’s trichrome and Elastin Verhoerff–van Gieson are available as Supplementary Figures S1 and S2, respectively. We retrieved Green Sea Turtle (*Chelodina mydas*) and Eastern long-necked turtle (*Chelodina longicollis*) H&E slides from Taronga Zoo (New South Wales, Australia) archives to compare valve-like structures in muscular arteries which had undergone the same routine processing.

A standard immunohistochemistry (IHC) protocol (Supplementary Material Section S1) was carried out for alpha smooth muscle actin (αSMA) as previously described [21]. Detection of smooth muscle was achieved using a mouse monoclonal primary antibody to a smooth muscle actin (αSMA) anti-human, Clone 1A4 (Agilent Technologies Inc., Code GA611, Santa Clara, CA, USA). 3,3′-diaminobenzidine (DAB) chromogen was utilised. To validate the antibody’s specificity, canine tissue was used as a positive control, and internal controls within the slide, such as vascular walls, were assessed. Negative controls included sections where the primary antibody was substituted with an isotype-matched control antibody at the same concentration, as well as sections where the primary antibody was omitted entirely, to ensure no non-specific binding from the secondary antibody or detection system. The absence of epithelial immunolabeling further confirmed the specificity of the αSMA staining (Supplementary Figure S3).

Elastin Verhoeff–van Gieson slides from a subset of *E. macquarii* turtles (*n* = 5) were scanned using an Olympus SLIDEVIEW^®^ VS200 slide scanner (Tokyo, Japan). Digital morphometry was performed using an Olympus OlyVIA^®^ (v4.2, Build 31689, Tokyo, Japan) and QuPath (v0.7.0, Belfast, Northern Ireland, UK) to calculate the linear or diameter-based and area-based percent of luminal narrowing, respectively. Scales were set to micrometres (µms). Only arteries sectioned in near-transverse orientation were included.

For linear-based measurements, the following parameters were recorded: total vessel diameter (D_total_; outer wall to outer wall), residual luminal diameter (D_lumen_; remaining open lumen), and maximal thickness of the valve-like structure (T_valve_; Supplementary Table S1). The latter was obtained by averaging the maximum length of each valve-like flap (Supplementary Table S3).

The percent luminal narrowing using the linear method was calculated as

$$\%\;\mathrm{luminal}\;\mathrm{narrowing}=\left(1-\frac{D_{lumen}}{D_{total}}\right)\times 100$$

Valve thickness ratio was calculated as

$$\frac{T_{valve}}{D_{total}}\times 100$$

For area-based measurements (µms^2^) and to confirm consistency of results, the outer vessel boundary and residual lumen were manually traced to obtain the total vessel area (A_total_), residual luminal area (A_lumen_) and the valve-like structure area (A_valve_; Supplementary Figure S4, Supplementary Table S2).

Area-based percent luminal narrowing was calculated as

$$\%\;\mathrm{luminal}\;\mathrm{narrowing}=\left(1-\frac{A_{lumen}}{A_{total\;vessel}}\right)\times 100$$

Valve area proportion was calculated as

$$\frac{A_{valve}}{A_{total\;vessel}}\times 100$$

Quantitative data were graphically represented using paired comparison plots comparing linear- and area-based estimates of luminal narrowing for each vessel, scatterplots evaluating the relationship between valve thickness ratio and area-based luminal narrowing, and the organ-based distribution plots of area-based luminal narrowing.

Descriptive statistics are presented as mean ± standard deviation (SD). Comparisons between linear- and area-based estimates were evaluated using a paired Wilcoxon signed-rank test. Correlations between valve thickness ratio and area-based luminal narrowing were assessed using Spearman’s rank correlation coefficient. Statistical analyses and figure generation were performed in Python (v. 3.11.2, Beaverton, OR, USA) using pandas, matplotlib, NumPy, and SciPy (Supplementary Material Section S2).

## 3. Results

All turtles were deemed clinically healthy and in optimal body condition. On histology, virtually all findings were considered either within normal limits or incidental except for three turtles in which tubulointerstitial lymphoplasmacytic inflammation in the kidneys was attributed to primitive cnidarian endoparasitic infection [23]. No intravascular or perivascular inflammation was noted, and there was no evidence of infection by intravascular trematodes (e.g., spirorchid parasites) or presence of perivascular trematode ova.

**Figure 1:**
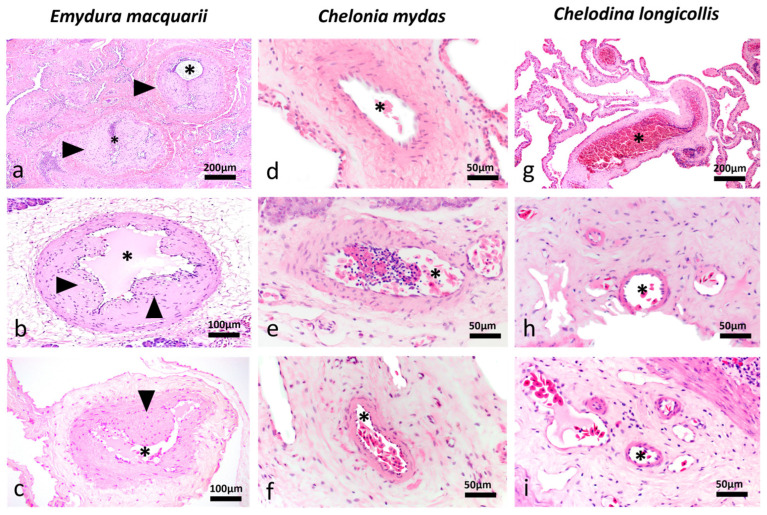
Valve-like structures in muscular arteries of a freshwater turtles, *Emydura macquarii*, in comparison to other diving reptiles. (**a**–**c**) Macquarie River Turtle, *Emydura macquarii*. (**a**) Lung, haematoxylin and eosin—H&E, 10×, (**b**) pancreas, H&E, 20×, (**c**) gastrointestinal tract—GIT, adventitial oesophageal artery, H&E, 20×. Muscular arteries show symmetrical valve-like structures (arrowheads) which project into narrowed vascular lumens (asterisks). (**d**–**f**) Green Sea Turtle, *Chelonia mydas*. (**d**) Lung, H&E, 40×, (**e**) pancreas, H&E, 40×, (**f**) GIT—small intestine, H&E, 40×. (**g**–**i**) Eastern long-necked turtle, *Chelodina longicollis*. (**g**) Lung, H&E, 10×, (**h**,**i**) GIT, upper GIT/oesophagus, H&E, 40×. Muscular arteries in the corresponding organ systems of *Chelonia mydas* and *Chelodina longicollis* display open lumens (asterisks), and no comparable valve-like structures are observed in these deep- or shallow-diving taxa.

**Figure 2:**
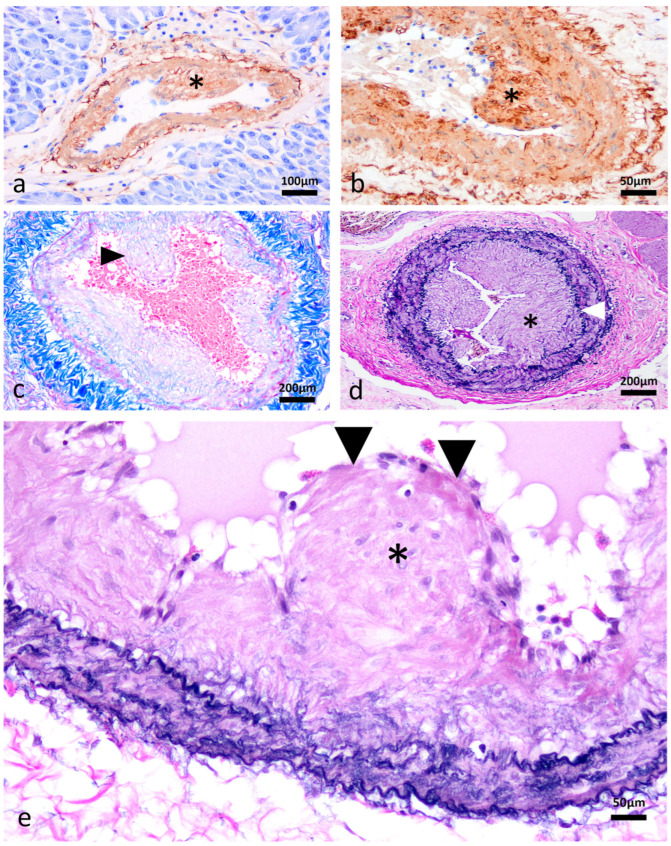
Immunohistochemistry (IHC) and histochemistry of vascular valve-like structures in muscular arteries of the Macquarie River Turtle, *Emydura macquarii*. (**a**,**b**) Pancreas. The valve-like projections of a pancreatic muscular artery are comprised entirely of smooth muscle. The endothelium is not immunoreactive (internal negative control). Alpha-smooth muscle actin—αSMA IHC, 20× and 40×, respectively. (**c**) Lungs. The prototypical faint red-magenta staining of the smooth muscle myocytes is observed forming the valve-like projections (black arrowhead), surrounded by markedly deep blue, adventitial collagen (internal positive control). Masson’s trichrome (MT), 10×. (**d**) Oesophagus. All valve-like structures (asterisk) are formed above the internal the elastic lamina (white arrowhead). Elastin van Gieson (EVG), 10×. (**e**) Pancreas. The valve-like structures expand the subendothelial space, denoting these valves as a type of neointimal formation. EVG, 40×.

**Figure 3:**
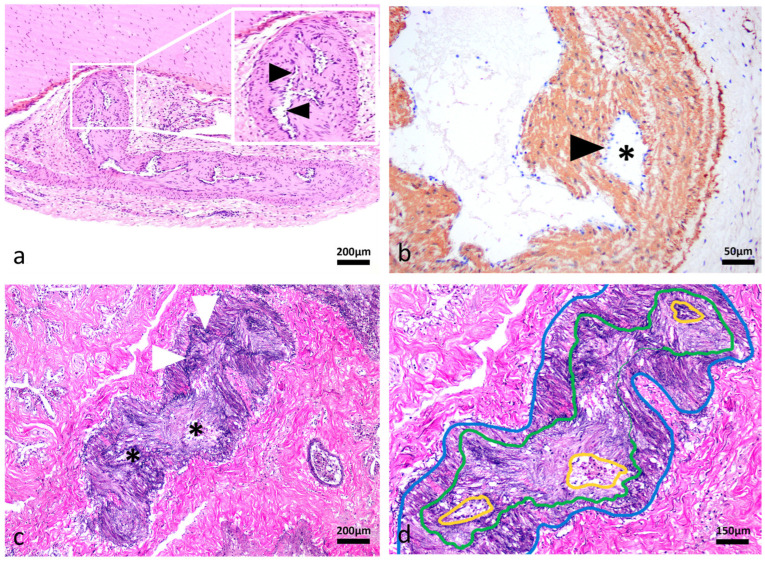
Intramural vascular channels in muscular arteries of a freshwater turtle, Macquarie River Turtle, *Emydura macquarii*. (**a**) Mesenteric muscular artery. The tunica media is irregularly thickened and frequently interrupted by multiple, discrete, slit-like vascular channels (black arrowheads). Haematoxylin and eosin (H&E), 10×. (**b**) Mesenteric muscular artery. Slit-like channels (asterisk) are supported by well-organised smooth muscle myocytes and are lined by endothelium (black arrowhead), in contrast to disorganised arrays of smooth muscle myocytes seen in pathologic conditions. Alpha smooth muscle actin (αSMA) immunohistochemistry, 40×. (**c**) Mesenteric muscular artery. The multiple slit-like channels (asterisks) are supported by organised smooth muscle myocytes above the internal elastic lamina (white arrowheads), Elastin van Gieson (EVG), 10×. (**d**) Mesenteric muscular artery. All normal layers are present, including tunica adventitia (blue line), internal elastic lamina (green line), and a vascular channel lined by endothelium (yellow line), EVG, 15×.

**Figure 4:**
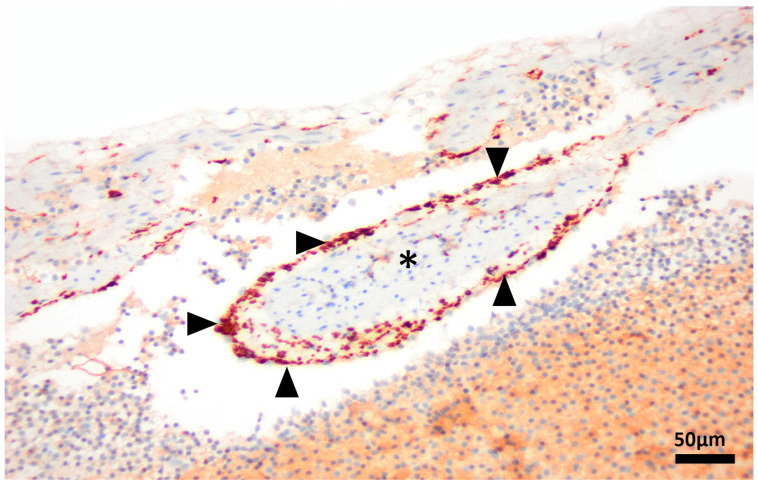
Cardiac atria of freshwater turtles, Murray River Turtle, *Emydura macquarii*. Pectinate muscle (asterisk). Numerous alpha smooth muscle actin (αSMA)-positive cells, identified as smooth muscle myocytes (arrowheads), are observed discontinuously lining a single atrial pectinate muscle. αSMA IHC, 40×.

Histological analysis revealed segmental, symmetrical, subintimal thickening mimicking valves within medium- and large-calibre muscular arteries in all major organ systems in 13/13 turtles leading to variable degrees of luminal narrowing (Figure 1a–c), in comparison to normal tissue of *C. mydas* (Figure 1d–f) and *C. longicollis* (Figure 1g–i). These structures appear to occur segmentally and not throughout the entire length of muscular arteries. Conventional H&E, histochemistry staining and IHC methods revealed the valve-like projections comprised smooth muscle myocytes that expanded the vascular wall (Figure 2a–c), always above the internal elastic lamina (Figure 2d,e). In 4/13 turtles, the subintimal tissue proliferation was interrupted by endothelium-lined cavitations (Figure 3a–d). Additionally, αSMA IHC was applied to 10/13 cardiac atria to determine the presence of smooth muscle in the endothelial lining (Figure 4).

**Figure 5:**
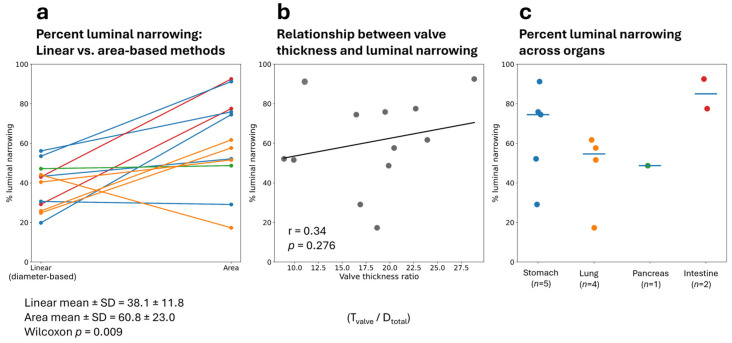
Quantitative morphometric analysis of valve-like intraluminal structures in arteries of *Emydura macquarii.* (**a**) Paired comparison of percent luminal narrowing estimated using linear (diameter-based) and area-based morphometric methods for individual vessels. Area-based measurements consistently yielded higher estimates of luminal compromise than linear measurements. Each line represents vessels in the stomach (blue), lung (orange), pancreas (green), and intestine (red). (**b**) Scatterplot illustrating the relationship between valve thickness ratio (T_valve_/D_total_) and area-based percent luminal narrowing. A weak positive correlation was observed between protrusion thickness and degree of luminal reduction. Grey circles represent individual vessels, and the black line represents the fitted linear regression trendline. (**c**) Distribution of area-based percent luminal narrowing across organ systems, including stomach, lung, pancreas, and intestine. Coloured circles follow the same organ-system colour scheme used in Panel (a). Horizontal blue bars indicate median values.

Area-based narrowing ranges were between 17.3% to 92.8%, with most values clustering between 50- and 80-based measurements, producing higher estimates of luminal narrowing than linear (diameter-based) measurements. Mean linear-based narrowing was approximately 38%, whereas mean area-based narrowing was approximately 61%. The paired Wilcoxon signed-rank test demonstrated a statistically significant difference between the two approaches (*p* = 0.009; Figure 5a). A weak-to-moderate positive relationship between valve thickness ratio and area-based luminal narrowing (Spearman r = 0.34) is observed in Figure 5b, although this correlation is not statistically significant (*p* = 0.276). There was variability observed in luminal narrowing across organ systems, with the highest degrees of luminal reduction observed in intestinal and some gastric vessels (Figure 5c).

## 4. Discussion

This study highlights the structural complexity of the cardiovascular system in *Emydura macquarii*, a semi-aquatic, shallow-diving freshwater turtle. Our observations revealed valve-like structures projecting into the lumens of muscular arteries across physiologically dynamic organs, such as respiratory and gastrointestinal vasculature. These structures were typically bilaterally symmetrical and composed of well-organised, mature smooth muscle myocytes, suggesting they represent normal anatomical features rather than pathological or artefactual changes. The valve-like structures were observed along both small- and medium-sized muscular arteries, forming slit-like protrusions into the vessel lumen without fully obstructing flow. To our knowledge, such structures have not been described previously in Chelid turtles.

The localization of these valve-like structures along muscular arteries aligns with the expected physiology of vascular smooth muscle. Muscular arteries contain a predominance of contractile cells in the tunica media, which support regulation of local blood flow under moderate pressure gradients, whereas elastic arteries rely more on elastic fibres to withstand high-pressure pulsatile flow [24]. The composition of these valve-like structures of organised smooth muscle myocytes suggests potential for localised modulation of blood flow or luminal diameter, although functional testing is required for confirmation. Similarly to previously described in other vertebrates, turtle arteries are composed of thick muscular and elastic layers to direct cardiac output, while veins have thinner tunica media and often contain valves that prevent backflow in low-pressure systems [24,25,26]. Valves have also been documented in the veins of sea turtles [27], highlighting the potential significance of venous structures in diving physiology, a fact yet to be confirmed in freshwater Chelid turtles.

Alternative explanations for the valve-like structures were considered. Pathological processes such as thrombosis, atherosclerosis, or hypertensive remodelling—such as endothelial dysfunction and vascular wall thickening—were not supported by the histological evidence [28]. The valve-like structures were consistent across multiple individuals, symmetrical, and lacked excessive extracellular matrix or fibrotic tissue. Inflammatory changes were absent in vascular and perivascular tissue, no evidence of nephrosclerosis, hyaline arteriosclerosis was observed in any turtle, and renal histology was unremarkable in most turtles [29,30], supporting the interpretation of these structures as normal anatomical features and not isolated local lesions, post-mortem vascular collapse changes or artefacts. These structures were not observed along the entire length of all muscular arteries in all turtles. In other words, if an individual had valve-like structures in pancreatic vessels, it might not have shown the same finding in the intestines; however, the opposite would be found in another turtle. As this finding was made opportunistically, we cannot verify the extent, size and exact vessels displaying this feature. Although the presence of multiple lumens within some vessels superficially resembles recanalized thrombi, these structures lacked endothelial disruption, fibrosis, or luminal obstruction typical of thrombosis. Likewise, they differ from venous sinuses, which are valveless and limited to venous beds [31].

The observed valve-like structures may also contribute to redistribution of blood flow or regulation of oxygen delivery during submergence. Comparative studies in reptiles have demonstrated that structural specialisation of the heart can facilitate functional separation of blood flow, including in species with a functionally divided ventricle [32], supporting the concept that anatomical features may contribute to regulated intracardiac flow dynamics. As freshwater turtles undergo peripheral vasoconstriction and bradycardia during dives [33,34], localised modulation of luminal diameter via smooth muscle protrusions could theoretically optimise tissue perfusion and maintain adequate oxygenation, particularly in smaller three-chambered hearts. Partial pressure of oxygen is not consistent during dives, as determined experimentally in aquatic turtles [35,36], suggesting that vertebrates capable of diving need different oxygen saturation transport mechanisms to ensure proper tissue oxygenation. While *E. macquarii* are shallow divers with limited capacity for aquatic respiration (river depths averaging 3–5 m) [7,37] and unlikely to experience extreme hydrostatic pressures, subtle flow regulation may still provide physiological benefit during routine submergence. As revealed via immunolabeling of αSMA—an important contractile cytoplasmic protein of smooth muscle myocytes—we have confirmed the presence of a monolayer of smooth muscle lining the atria of *E. macquarii*. Smooth muscle has also been previously reported to line cardiac atrium and sinus venosus endocardium in Emydid turtles [38]. Speculation on function has been associated with diving physiology or as a regulator of cardiac output. In this context, atrial smooth muscle may also contribute to modulation of ventricular filling dynamics, potentially supporting reduced cardiac output and bradycardia during prolonged apnoea, as suggested in previous studies [38,39]. The presence of non-vascular smooth muscle within the heart has been confirmed in eight phylogenetically diverse Pleurodiran and Cryptodiran turtle species (*Trachemys scripta* [Emydidae]; *Chelonoidis carbonarius* and *Testudo hermanni* [Testudinae]; *Chelydra serpentina* [Chelydridae]; *Cyclanorbis senegalensis* [Cyclanorbinae]; *Pelodiscus sinensis* [Trionychidae]; *Pelomedusa subrufa* [Pelomedusidae]; and *Chelodina mccordi* [Chelidae]) [38]. These species are all aquatic turtles, except for the Testudinae (tortoises), which evolved relatively recently from aquatic ancestors [40], underpinning the argument that retention of these vascular anatomical features across taxa may still be possible despite phylogenetic divergence.

The significance of the persistence of smooth muscle in several freshwater turtle species in the sinus venosus throughout evolution in multiple and phylogenetically distant taxa remains undetermined [39,41]. These findings suggest that the distribution and contractile function of atrial smooth muscle in turtles may contribute to the formation of a functional barrier against the mixing of oxygenated and de-oxygenated blood, as seen in non-crocodilian reptile species carrying a single, partially septated cardiac ventricle [42,43]. The presence of non-vascular smooth muscle within the atrial myocardium in *E. macquarii*, a Chelid turtle, demonstrates comparable structures to those documented for Emydid turtles. These two taxa, however, are phylogenetically distant, belonging to two major extant turtle clades, Pleurodira and Cryptodira, respectively, which have been separated by over 160 million of years of evolution [40,44]. The presence of similar atrial structures in distantly related turtle families may therefore reflect either convergent evolution associated with shared functional demands of aquatic cardiovascular physiology or the retention of ancestral morphological traits within Testudines. Turtles have a long evolutionary history in aquatic ecosystems, and it is conceivable that such structures represent ancient features retained across divergent lineages. Resolving whether these similarities represent convergence or deep ancestral homology will require broader comparative investigation across additional turtle taxa.

The structural organisation of smooth muscle myocytes is integral to maintaining vascular integrity and function [45]. In healthy vessels, vascular smooth muscle cells are arranged in a highly organised manner, supporting slit-like channels lined by endothelium [46]. This orderly arrangement facilitates efficient contractile function and blood flow regulation [47]. In Chelonians, the presence of smooth muscle myocytes on the surface of atrial pectinate muscles is atypical, and disorganisation may indicate pathological remodelling of histological features. While atrial smooth muscle has been identified in various Testudines as outlined above, this feature is not universally present across all turtle species. For instance, its absence in the loggerhead sea turtle (*Caretta caretta*) suggests species-specific variation [41]. In *E. macquarii*, the additional layer of atrial smooth muscle myocytes may support flow of oxygenated blood preferentially into the cavum arteriosum, the exiting chamber of the turtle ventricle. Another alternative explanation would be that the atrial endothelium is immunoreactive or cross-reactive for αSMA. However, we have demonstrated that the endothelium of muscular arteries in *E. macquarii* is negative for αSMA using internal negative controls in IHC, as documented for muscular arteries, such as coronary arteries in humans [48].

As ectothermic vertebrates, turtles are dependent on environmental temperatures for regulation of their body temperature [49]. A previous study in freshwater turtles [50] suggested that chronic exposure to low temperatures (~5 °C) can modify cardiac shunting patterns, increasing net shunt flows through reduced systemic resistance and higher total systemic blood flow. In the population studied here and in recent southern Queensland history, these turtles have inhabited sites without prolonged water temperatures below 5 °C (pers. comm. S. Vardy). Nonetheless, Chelid turtles are a lineage with a long evolutionary history associated with cooler climates [51], originating within Gondwanan systems that included regions such as South America and Antarctica [52,53]. Throughout their evolutionary history, these turtles likely experienced repeated exposure to colder environmental conditions, including those associated with past glacial periods [54], even though Australia itself remained largely ice-free [55]. Consistent with this history, several Chelid taxa occupy cool, oxygen-rich aquatic habitats, and some species exhibit specialised physiological adaptations such as cloacal ventilation that are associated with cold environments [56]. In comparison with other Australian Chelids, *E. macquarii* is quite mobile and spends prolonged periods near the water surface [57,58], with a higher rate of oxygen consumption during exercise [57]. In aquatic tetrapods, coelomic and vascular structural adaptations are commonly associated with environmental pressures such as temperature and oxygen availability, reflecting patterns of physiological adaptation [59]. Within this broader evolutionary and physiological context, the valve-like vascular structures observed here are most appropriately interpreted as features developed in response to historic temperature-related, metabolic and exertion-based vascular remodelling.

Intramural vascular elaboration in aquatic and semi-aquatic vertebrates has been most clearly demonstrated in large elastic arteries and specialised oxygen delivery systems, where it is associated with enhanced metabolic support and haemodynamic adaptation. Although these vertebrates exhibit diverse vascular adaptations to environmental pressures, direct evidence connecting intramural vascular channels in small to medium-calibre arteries with specialised vasa vasorum arrangements remains limited. In the hooded seal (*Cystophora cristata*), a highly developed vasa vasorum interna penetrates the full thickness of the ascending aorta, likely maintaining perfusion pressure during prolonged diastole, thereby reducing cardiac workload during diving [60]. Similarly, extensive vascular networks in fish gills and air-breathing organs, such as those described in the climbing perch (*Anabas testudineus*), reduce diffusion distances, and while not strictly vasa vasorum, demonstrate how intramural capillary networks can be elaborated to reduce diffusion distances and optimise oxygen delivery [61]. Collectively, these observations suggest that intramural vascular channels may represent a continuum of vasa vasorum elaboration in response to combined hypoxic and haemodynamic factors. However, direct evidence for such specialised arrangements in small to medium-calibre systemic arteries remains limited, and their interpretation as modified vasa vasorum in Chelonians should therefore be considered provisional pending further structural and functional validation.

The valve-like structures were associated with substantial luminal narrowing. Area-based morphometry consistently estimated greater luminal reduction than linear methods, with linear measurements ranging from approximately 20 to 55% compared with area-based estimates that frequently exceeded 50% and occasionally surpassed 90%. This discrepancy likely reflects the highly irregular, often stellate configuration of the residual lumen, meaning that single linear diameter measurements underestimate the true reduction in luminal cross-sectional area. Accordingly, area-based measurements likely provide a more physiologically relevant estimate of luminal compromise in these vessels.

Valve-like structure thickness scaled imperfectly with luminal narrowing, indicating that larger protrusions did not always produce greater luminal reduction. Some vessels with moderate T_valve_ values exhibited severe area narrowing, whereas others with larger protrusions retained greater luminal space. These findings suggest that overall geometry and configuration of the intraluminal structures are more important determinants of luminal compromise than protrusion thickness alone, supporting the interpretation that these are complex intraluminal folds or projections rather than simple concentric mural thickening.

Collectively, the morphometric data demonstrate that these structures can occupy a substantial proportion of the vascular lumen and support the interpretation that they represent prominent anatomical features rather than minor mural irregularities or sectioning artefacts. Although intestinal and gastric vessels appeared to exhibit greater luminal reduction, the limited sample size and unequal organ representation preclude conclusions regarding organ-specific differences.

Two outliners (Turtle ID 1 and 2) were found in the valve-like structure linear and area-based measurements. Turtle ID 1 had a valve area proportion of 0.09% paired with an area narrowing of 92.77% and Turtle ID 2 had a 71.8% narrowing but only a 3.74% valve area proportion, which are mathematically inconsistent. After revising that the luminal tracing, annotation, vessel boundary delimitation were correct, and that there were no unit conversion issues, a decision was made to exclude these two from the overall area-proportion analysis. Data from these two turtles is still provided in Supplementary Tables S1–S3.

Recent advances in aquatic animal post-mortem imaging and virtual necropsy provide relevant methodological context for future anatomical studies in reptiles. Standardised ultrasonographic and computed tomography approaches, together with multi-modal imaging and post-processing techniques, have been applied across a range of aquatic taxa, to improve coelomic assessment, morphometric standardisation, and post-mortem diagnostic evaluation [62,63,64,65,66,67]. While not employed in the present study, these approaches may provide useful complementary tools for future investigations of the functional significance of the observed projections in *E. macquarii.* Furthermore, a key limitation of this study is the exclusive use of adult female specimens. While this provides a consistent baseline for morphological assessment, potential sexual dimorphism in cardiovascular structure and function cannot be excluded, due to possible sex-related variation [68]. Future studies incorporating both sexes would be valuable to determine whether the observed features are sex-specific or broadly conserved within the species.

## 5. Conclusions

This study represents the first morphological description of two previously unclassified cardiovascular structures observed across multiple systems in several Chelid turtles: valve-like structures within muscular arteries and the presence of atrial smooth muscle. We opportunistically gathered evidence that these are likely normal, rather than pathological, anatomical features. These findings expand the anatomical knowledge of turtle cardiovascular systems, underpinning previously unrecognised complexity in reptilian vascular regulation. The evolutionary pressure leading to the formation of such anatomical features and their exact purpose remain unknown. However, multiple compelling functional hypotheses are offered, noting the possible influence of historic cooler climates in shaping reptilian haemodynamics, as well as the relationship of these structures with peripheral blood flow regulation and submergence or postural physiology. The confirmation of the existence of atrial smooth muscle in an Australian Chelid turtle—previously reported among Pleurodirans only in *Chelodina mccordi* and *Pelomedusa subrufa* and observed here with more extensive coverage—may represent a convergent functional adaptation or a retained ancestral trait that provides solutions to aquatic life.

Further characterisation of haemodynamic function, contractility and effects on vascular resistance or flow direction of the valve-like structures, via microangiography, in vivo Doppler flow studies or vascular perfusion experiments, is instrumental in determining the physiological role of these structures. Additional developmental and histological investigations into the atrial smooth muscle would assist in determining their timing of development and molecular pathways driving their formation and, ultimately, their potential role in influencing postural and diving reptile haemodynamics.

## Data Availability

Raw data from linear and area-based measurements taken for digital morphometry, as well as the Python code ran for statistical analyses and graphical representation of results, have been included as Supplementary Materials. Pathology reports with the histopathology are contained within clinical medical records that include confidential client information. In accordance with The University of Queensland’s Veterinary Laboratory Services policies and procedures, these records cannot be made publicly available.

## Supplementary Materials

The following supporting information can be downloaded at https://www.mdpi.com/article/10.3390/vetsci13050493/s1, Figure S1: Investigation into Masson’s trichrome stain in *Emydura macquarii* turtles; Figure S2: Investigation into Elastin Verhoerff–van Gieson stain in *Emydura macquarii* turtles; Figure S3: Investigation into the immunolabeling of smooth-muscle actin (cytoplasmic) marker in *Emydura macquarii* turtles in contrast with canine control tissue; Material Section S1: Immunohistochemistry protocol for alpha smooth muscle actin (αSMA) marker applied to Murray River Turtles (*Emydura macquarii*); Figure S4: Schematic workflow for linear and area-based morphometric analysis of valve-like intraluminal arterial structures in Murray River Turtles (*Emydura macquarii*); Material Section S2: Python code used for statistical analyses and graphical representation of vascular morphometric data from valve-like structures in Murray River Turtles (*Emydura macquarii*). Table S1: Raw linear or diameter-based morphometric measurements of valve-like intraluminal structures identified in arterial sections of Murray River Turtles (*Emydura macquarii*); Table S2: Raw area-based morphometric measurements of valve-like intraluminal structures identified in arterial sections of Murray River Turtles (*Emydura macquarii*); Table S3: Raw linear valve-like structure thickness measurements from muscular arteries in various systems in Murray River Turtles (*Emydura macquarii*).

## Abbreviations

DES	Queensland Department of Environment and Science
DETSI	Queensland Department of Environment, Tourism, Science and Innovation
CSIRO	Commonwealth Scientific and Industrial Research Organisation
AEC	Animal Ethics Committee
H&E	Haematoxylin and eosin
MT	Masson’s trichrome
IHC	Immunohistochemistry
αSMA	alpha smooth muscle actin
EVG	Elastin van Gieson
GIT	Gastrointestinal tract
