# Supplementary material for: Morphological Investigation of Uncharacterised Cardiovascular Structures in Shallow-Diving, Semi-Aquatic Freshwater Turtles (Chelidae: Emydura macquarii)
Source: Vet Sci. 2026 May 19;13(5):493. doi: 10.3390/vetsci13050493 (PMC13211658; doi:10.3390/vetsci13050493)
Supplement: Supplementary file 1 [file vetsci-13-00493-s001.zip › vetsci-4278696-supplementary.PDF]

Supplementary Material

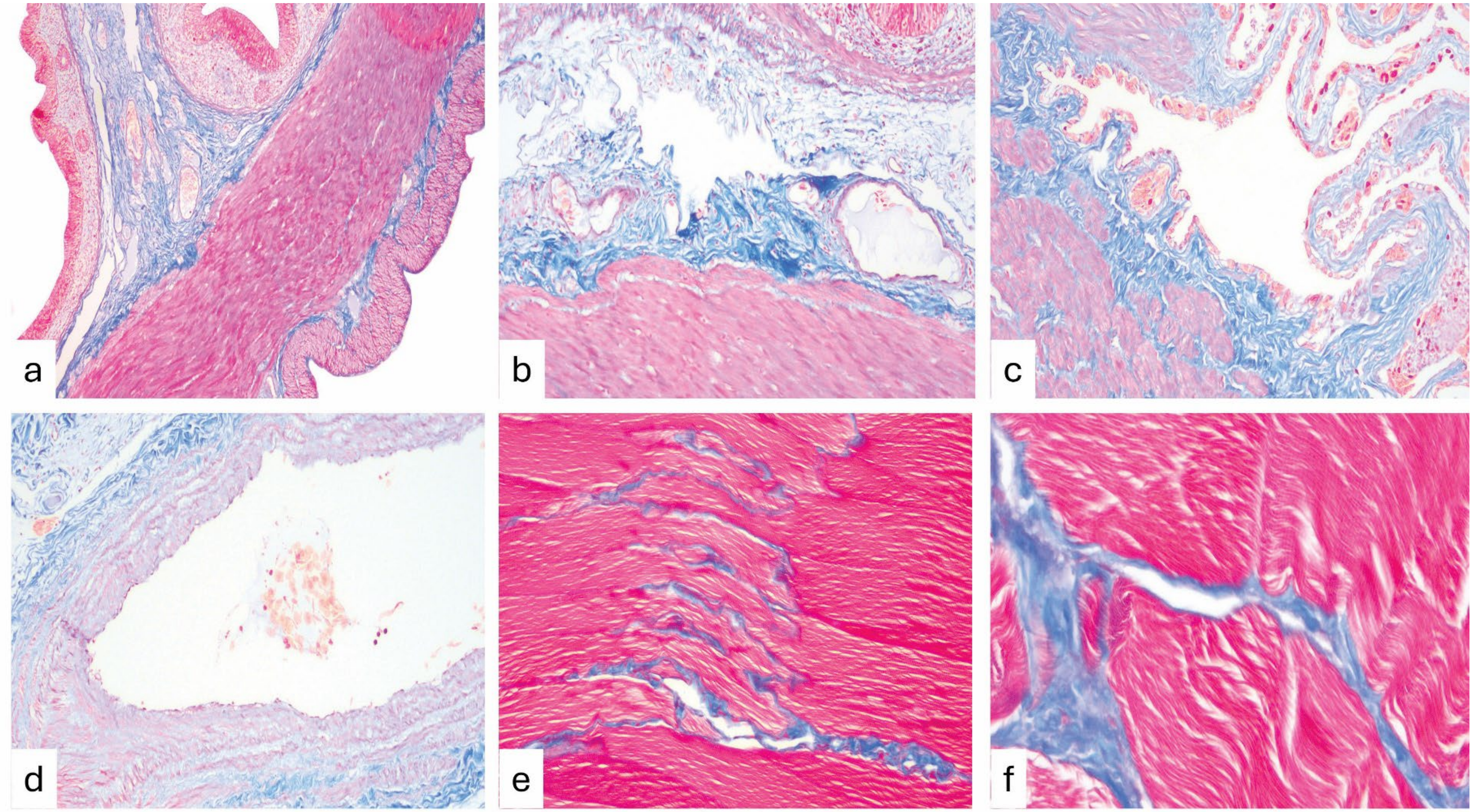

Supplementary Figure S1. Investigation into Masson's Trichrome stain in *Emydura macquarii* turtles. a-b) Intestine. Submucosal collagen fibers are stained blue, in contrast to the moderate red counter staining of the overlying epithelium and muscularis externa, 4x. **c) Lung**. The obliquus/transversus abdominis muscle are red, adventitial and stromal collagen fibers are blue and nucleated erythrocytes are pale orange-red as expected, 10x. **d) Pulmonary artery**. Smooth muscle myocytes in the adventitia stain pale red, contrasting to the deep blue collagen fibers of the perivascular adventitia, 10x. **e-f) Striated muscle**. The deep red counter stain highlights the muscular sarcoplasm, whilst the epimysial collagen is deep blue, evident at 10x (e) and 40x (f).

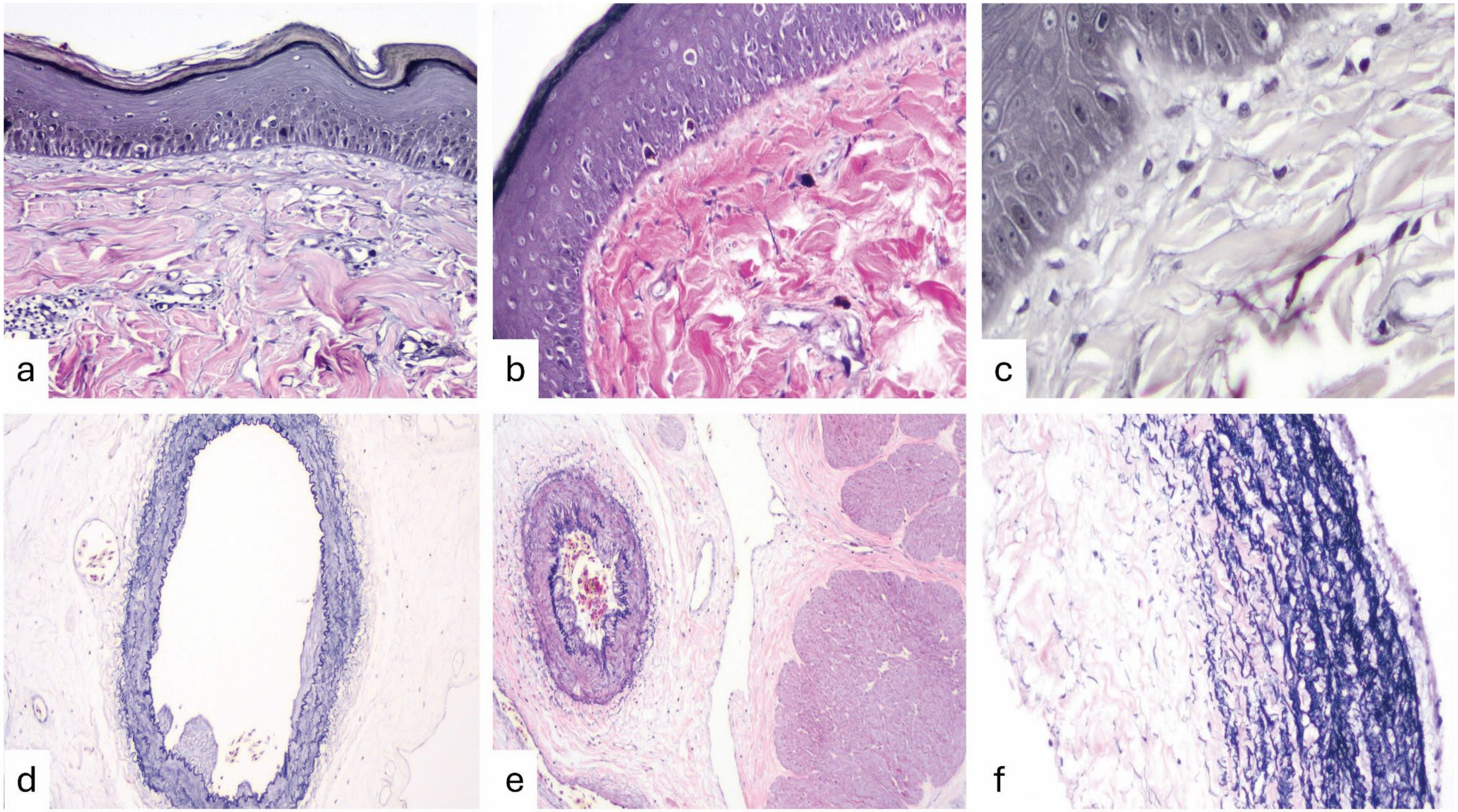

Supplementary Figure S2. Investigation into Elastin Verhoeff-van Gieson stain in *Emydura macquarii* turtles. a-c) Skin. Elastin tissue is observed interspersed between normal mature collagen bundles in the extracellular matrix, at 10x (a), 20x (b), and 40x (c) magnifications. **d-f) Arteries**. Elastic fenestrated lamellae are observed within the tunica media of arteries, arranged in concentric rings between smooth muscle myocytes as seen at 4x (d), 10x (e), and 20x (f).

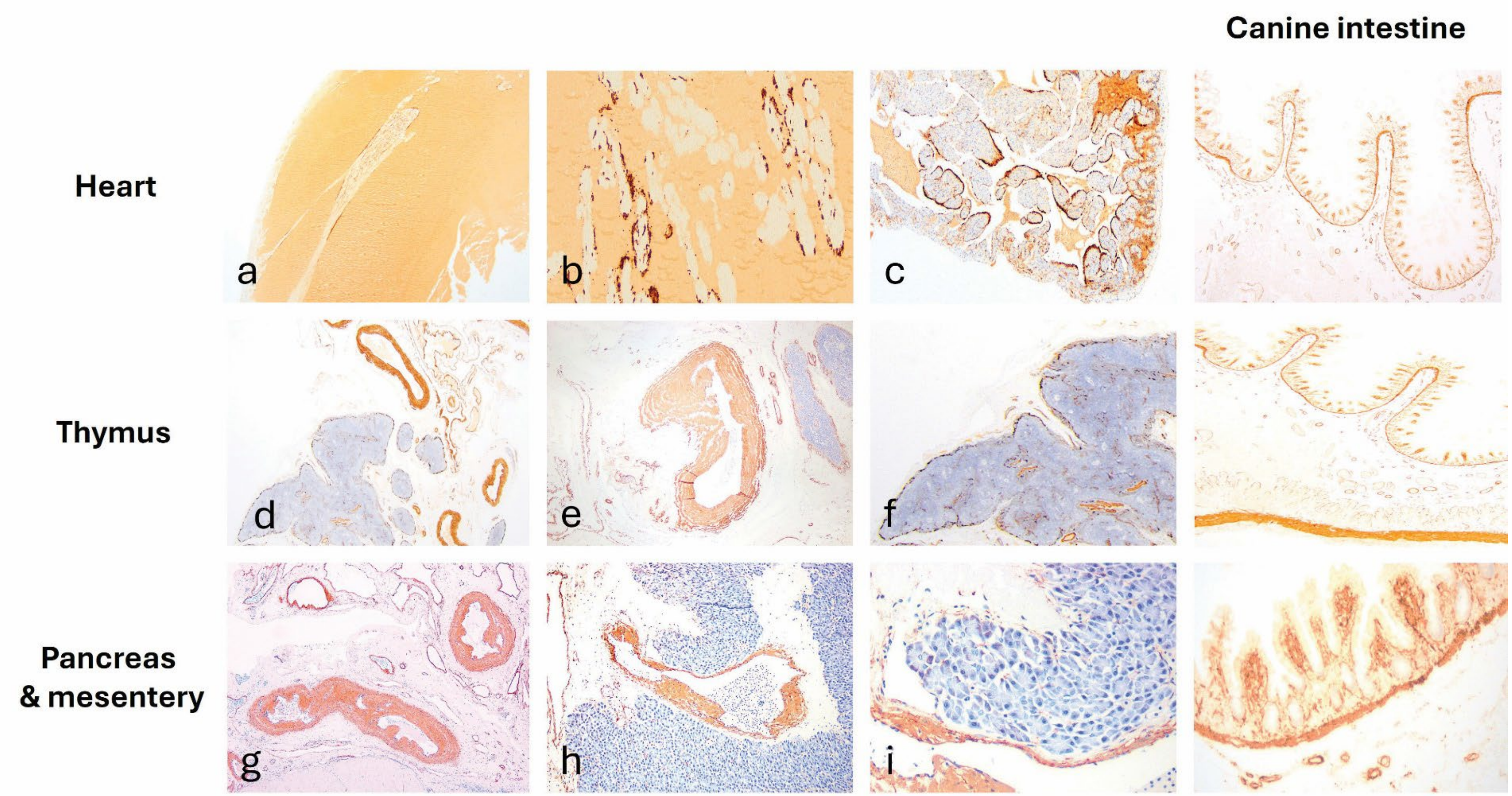

Supplementary Figure S3. Investigation into the immunolabeling of smooth-muscle actin (cytoplasmic) marker in *Emydura macquarii* turtles in contrast with canine control tissue. a-c) Heart. The distribution of smooth-muscle actin is noted selectively lining the pectinate musculature within cardiac atria, evident at 1.25x (a), 10x (b) and 4x (c) magnifications. Note the negative immunolabeling of striated cardiac musculature**. d-f) Thymus and adjacent large caliber muscular arteries.** The positive immunolabeling is observed within the cytoplasm of smooth muscle myocytes comprising the muscular arterial wall. Adjacent stroma and thymic lymphoid tissue does not show labeling at 1.25X (d), 4x (e), and 10x (f). **h-i) Pancreas and mesenteric vasculature**. Immunolabeling is appreciated within the tunica media of muscular arteries and veins. The vascular adventitia, adjacent stroma and pancreatic acini are negative. All canine controls show normal cytoplasmic labeling of villar smooth muscle, muscularis mucosa, and small caliber submucosal vasculature.

Supplementary Material Section S1. Immunohistochemistry protocol for alpha smooth muscle actin (αSMA) marker applied to Murray River Turtles (*Emydura macquarii*).

Immunohistochemistry for smooth muscle actin (SMA; Clone 1A4) was performed on tissue samples displaying stromal or vascular smooth muscle involvement. IHC was carried out using the DAKO OMNIS Automatic Stainer with 4-µm sections mounted on DAKO IHC-coated slides, following a 30-minute heat fixation step at 95°C to promote tissue adhesion. The SMA antibody was used as a commercially available pre-dilution (Clone 1A4, Agilent Technologies, Santa Clara, CA, USA). Chromogenic detection was achieved using the DAKO EnVision™ FLEX High pH Detection Kit, which includes HRP-conjugated secondary reagents and DAB+ chromogen. Protocol steps adhered to the manufacturer's instructions and institutional standards for validated internal controls. Tissues were selected based on histological features suggestive of smooth muscle differentiation, and staining was evaluated in conjunction with histopathological findings to confirm smooth muscle lineage.

1. Clearify 1min
2. Clearify 1min
3. DI Water Wash 5 sec
4. 95°C High pH (pH 9) Heat Induced Epitope Retrieval (HIER) 30 min
5. Wash Buffer 3 min
6. SMA Antibody 20 min
7. Wash Butter 2 min
8. Peroxidase Blocking Reagent 3 min
9. Wash Buffer 2 min
10. Horse Radish Peroxidase (HRP) 20 min
11. Wash Buffer 2 min
12. Wash Buffer 2 min
13. DI Water 30 sec
14. Wash Buffer 2 min
15. DAB Working Sol. 5 min
16. Wash Buffer 2 min
17. DI Water 30 sec
18. Wash Buffer 2 min
19. Haematoxylin 3 min
20. DI Water 2 min
21. Wash Buffer 2 min
22. Dehydrate, Clear and Mount.

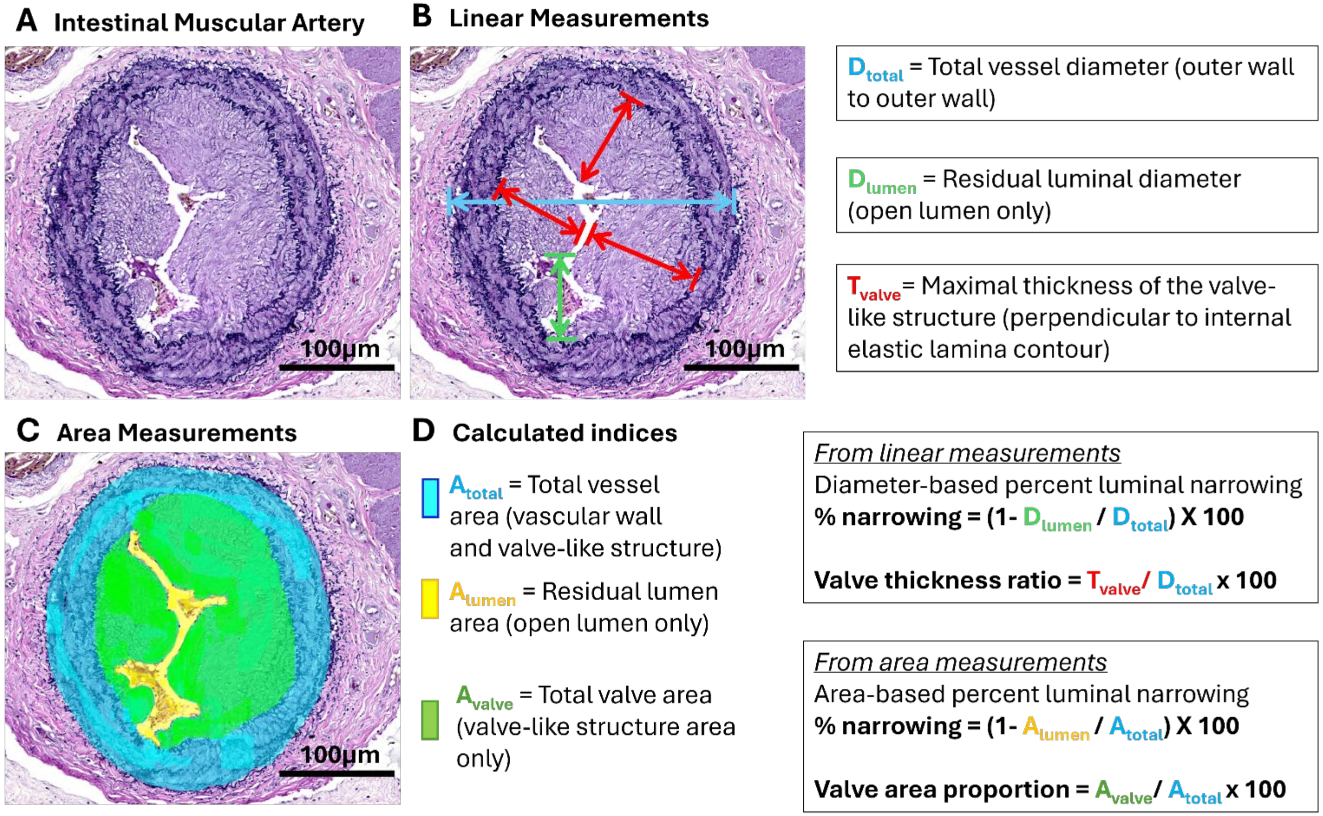

Supplementary Figure S4. Schematic workflow for linear- and area-based morphometric analysis of valve-like intraluminal arterial structures in Murray River Turtles (*Emydura macquarii*). A) Representative EVG-stained arterial section containing a valve-like intraluminal structure prior to annotation or morphometric analysis. B) Linear (diameter-based) morphometric measurements performed on the same vessel section. Measurements included total vessel diameter (D_{total}; outer wall to outer wall), residual luminal diameter (D_{lumen}; maximal residual open lumen), and maximal valve-like structure thickness (T_{valve}; maximal protrusion from the expected inner vascular contour into the lumen). C) Area-based morphometric annotations of the same vessel section. Manual tracings were used to determine total vessel area (A_{total}), residual luminal area (A_{lumen}), and valve-like structure area (A_{valve}).

Supplementary Material Section S2. Python code used for statistical analyses and graphical representation of vascular morphometric data from valve-like structures in Murray River Turtles (*Emydura macquarii*).

```
from io import BytesIO
import pandas as pd
import matplotlib.pyplot as plt
import numpy as np
from scipy.stats import wilcoxon, spearmanr

# Data
data = {
  "turtle_ID":[3,4,4,4,4,5,5,5,5,5,5,5],
  "organ_system":["Intestine","Stomach 1","Stomach 2","Stomach 3","Intestine",
          "Stomach1","Stomach2","lung","Pancreas","Lung1","Lung2","Lung3"],

"linear_narrowing":[29.20,19.80,43.26,56.13,43.01,53.50,30.59,44.04,47.15,24.85,25.8
3,40.39],

"valve_thickness_ratio":[22.71,16.47,8.88,19.50,28.86,11.04,16.89,18.65,19.87,20.44,
23.93,9.91],

"area_narrowing":[77.50,74.50,52.13,75.87,92.49,91.20,29.08,17.29,48.71,57.63,61.71
,51.58]
}
df = pd.DataFrame(data)
# Stats
w_stat, w_p = wilcoxon(df["linear_narrowing"], df["area_narrowing"])
rho, p_corr = spearmanr(df["valve_thickness_ratio"], df["area_narrowing"])
```

```
# Organ grouping cleanup
def organ_group(x):
    x = x.lower()
    if "stomach" in x:
        return "Stomach"
    if "lung" in x:
        return "Lung"
    if "pancreas" in x:
        return "Pancreas"
    if "intestine" in x:
        return "Intestine"
    return x

df["organ_group"] = df["organ_system"].apply(organ_group)

# Create figure
fig = plt.figure(figsize=(16,6))

# Panel A
ax1 = plt.subplot(1,3,1)

for i in range(len(df)):
    ax1.plot([0,1], [df.loc[i,"linear_narrowing"], df.loc[i,"area_narrowing"]],
             marker='o')

ax1.set_xticks([0,1])
ax1.set_xticklabels(["Linear\n(diameter-based)", "Area\n(area-based)"])
ax1.set_ylabel("% luminal narrowing")
```

```
ax1.set_ylim(0,100)
ax1.set_title("A\nPercent luminal narrowing:\nlinear vs. area-based methods",
          loc='left', fontsize=14, fontweight='bold')

text = (
   f"Linear mean ± SD = {df['linear_narrowing'].mean():.1f} ±
{df['linear_narrowing'].std():.1f}\n"
   f"Area mean ± SD = {df['area_narrowing'].mean():.1f} ±
{df['area_narrowing'].std():.1f}\n"
   f"Wilcoxon p = {w_p:.3f}"
)
ax1.text(0.02, -0.28, text, transform=ax1.transAxes, fontsize=10)

# Panel B
ax2 = plt.subplot(1,3,2)

x = df["valve_thickness_ratio"]
y = df["area_narrowing"]

ax2.scatter(x, y)

coef = np.polyfit(x, y, 1)
poly1d_fn = np.poly1d(coef)

x_line = np.linspace(x.min(), x.max(), 100)
ax2.plot(x_line, poly1d_fn(x_line))

ax2.set_xlabel("Valve thickness ratio\n(T_valve / D_total)")
ax2.set_ylabel("% luminal narrowing (area-based)")
```

```
ax2.set_ylim(0,100)
ax2.set_title("B\nRelationship between valve thickness\nand luminal narrowing",
          loc='left', fontsize=14, fontweight='bold')

ax2.text(0.05, 0.90,
        f"Spearman r = {rho:.2f}\np = {p_corr:.3f}",
        transform=ax2.transAxes,
        fontsize=11)

# Panel C
ax3 = plt.subplot(1,3,3)

groups = ["Stomach", "Lung", "Pancreas", "Intestine"]
positions = np.arange(len(groups))

for pos, grp in zip(positions, groups):
    vals = df[df["organ_group"] == grp]["area_narrowing"]
    xj = np.random.normal(pos, 0.04, size=len(vals))
    ax3.scatter(xj, vals)
    if len(vals) > 0:
        med = np.median(vals)
        ax3.hlines(med, pos-0.2, pos+0.2, linewidth=2)

ax3.set_xticks(positions)
ax3.set_xticklabels([f"{g}\n(n={len(df[df['organ_group']==g])})" for g in groups])
ax3.set_ylabel("% luminal narrowing (area-based)")
ax3.set_ylim(0,100)
ax3.set_title("C\nPercent luminal narrowing across organs",
```

```
         loc='left', fontsize=14, fontweight='bold')

plt.tight_layout()

outpath = "/mnt/data/Figure2_quantitative_morphometry.png"
plt.savefig(outpath, dpi=300, bbox_inches='tight')

print(outpath)
```

Supplementary Table S1. Raw linear or diameter-based morphometric measurements of valve-like intraluminal structures identified in arterial sections of Murray River Turtles *(Emydura macquarii)*.

		Area-based Data						Area-based Results	
Turtle ID	Organ system	Area_Wall (μm^2)	Area_Valve (μm^2)	Total_A_vessel (μm^2)	Total_A_vessel (mm^C)	A_Lumen (μm^2)	A_Lumen (mm^2)	% Narrowing	Valve area proportion
1	Stomach	1154444.78	1004.68	1155449.46	1.16	83556.28	0.084	92.77	0.09
2	Stomach	30629.31	1190.65	31819.96	0.03	9076.45	0.009	71.48	3.74
3	Intestine	31051.61	26439.73	57491.34	0.06	12937.55	0.013	77.50	45.99
4	Stomach 1	112652.75	61641.34	174294.10	0.17	44441.21	0.044	74.50	35.37
4	Stomach 2	149794.20	55543.94	205338.14	0.21	98293.86	0.098	52.13	27.05
4	Stomach 3	147374.79	73304.59	220679.38	0.22	53242.47	0.053	75.87	33.22
4	Intestine	85780.73	96426.45	182207.18	0.18	13676.27	0.014	92.49	52.92
5	Stomach 1	31924.09	26329.60	58253.68	0.06	5126.02	0.005	91.20	45.20
5	Stomach 2	14717.46	991.69	15709.15	0.02	11140.79	0.011	29.08	6.31
5	Lung	8940.93	5426.07	14367.00	0.01	11882.81	0.012	17.29	37.77
5	Pancreas	49351.28	64707.96	114059.23	0.11	58505.68	0.059	48.71	56.73
5	Lung 1	79229.55	88133.05	167362.60	0.17	70915.37	0.071	57.63	52.66
5	Lung 2	51122.56	60291.49	111414.05	0.11	42655.58	0.043	61.71	54.11
5	Lung 3	55101.13	84555.33	139656.46	0.14	67620.13	0.068	51.58	60.55

Supplementary Table S2. Raw area-based morphometric measurements of valve-like intraluminal structures identified in arterial sections of Murray River Turtles (*Emydura macquarii*).

		Linear-based Data			Linear-based Results	
Turtle ID	Organ system	D_total (μms)	D_lumen (μms)	T_valve (μms)	% Narrowing	Valve thickness ratio
1	Stomach	674	511	44	24.18	6.53
2	Stomach	290	139	37	52.07	12.76
3	Intestine	339	240	77	29.20	22.71
4	Stomach 1	601	482	99	19.80	16.47
4	Stomach 2	890	505	79	43.26	8.88
4	Stomach 3	718	315	140	56.13	19.50
4	Intestine	544	310	157	43.01	28.86
5	Stomach 1	471	219	52	53.50	11.04
5	Stomach 2	219	152	37	30.59	16.89
5	Lung	193	108	36	44.04	18.65
5	Pancreas	473	250	94	47.15	19.87
5	Lung 1	499	375	102	24.85	20.44
5	Lung 2	422	313	101	25.83	23.93
5	Lung 3	817	487	81	40.39	9.91

Supplementary Table S3. Raw linear valve-like structure thickness measurements from muscular arteries in various systems in Murray River Turtles (*Emydura macquarii*).

Turtle ID	Organ system	T_valve1 (µms)	T_valve2 (µms)	T_valve3 (µms)	T_valve4 (µms)	T_valve5 (µms)	T_valve_average (µms)
1	Stomach 1	51	52	45	44	27	43.8
2	Stomach 1	27	37	38	38	43	36.6
3	Intestine 1	75	87	95	52	-	77.25
4	Intestine 1	168	98	139	180	188	154.6
4	Stomach 1	78	94	72	86	164	98.8
4	Stomach 2	98	83	64	65	84	78.8
4	Stomach 3	36	124	215	183	-	139.5
5	Stomach 1	72	55	52	53	30	52.4
5	Stomach 2	37	38	32	37	41	37
5	Lung	40	34	41	32	32	35.8
5	Pancreas	58	80	145	102	85	94
5	Lungs 2	70	116	116	95	115	101
5	Lungs 3	72	83	89	170	99	102.2
5	Lungs 1	74	82	104	85	60	81
